# Generation of miniploid cells and improved natural transformation procedure for a model cyanobacterium *Synechococcus elongatus* PCC 7942

**DOI:** 10.3389/fmicb.2022.959043

**Published:** 2022-07-26

**Authors:** Sadaf Riaz, Ying Jiang, Meng Xiao, Dawei You, Anna Klepacz-Smółka, Faiz Rasul, Maurycy Daroch

**Affiliations:** ^1^School of Environment and Energy, Peking University Shenzhen Graduate School, Shenzhen, China; ^2^Department of Neuroscience, University of Connecticut Health Center, Farmington, CT, United States; ^3^Department of Bioprocess Engineering, Faculty of Process and Environmental Engineering, University of Technology, Łódź, Poland

**Keywords:** ploidy, miniploid, natural transformation, antioxidant, catalase, *Synechococcus*

## Abstract

The biotechnologically important and naturally transformable cyanobacterium, *Synechococcus elongatus* PCC 7942, possesses multiple genome copies irrespective of its growth rate or condition. Hence, segregating mutations across all genome copies typically takes several weeks. In this study, *Synechococcus* 7942 cultivation on a solid growth medium was optimised using different concentrations of agar, the addition of antioxidants, and overexpression of the catalase gene to facilitate the rapid acquisition of colonies and fully segregated lines. *Synechococcus* 7942 was grown at different temperatures and nutritional conditions. The miniploid cells were identified using flow cytometry and fluorimetry. The natural transformation was carried out using miniploid cells and validated with PCR and high performance liquid chromatography (HPLC). We identified that 0.35% agar concentration and 200 IU of catalase could improve the growth of *Synechococcus* 7942 on a solid growth medium. Furthermore, overexpression of a catalase gene enhanced the growth rate and supported diluted culture to grow on a solid medium. Our results reveal that high temperature and phosphate-depleted cells contain the lowest genome copies (2.4 ± 0.3 and 1.9 ± 0.2) and showed the potential to rapidly produce fully segregated mutants. In addition, higher antibiotic concentrations improve the selection of homozygous transformants while maintaining similar genome copies at a constant temperature. Based on our observation, we have an improved cultivation and natural transformation protocol for *Synechococcus* 7942 by optimising solid media culturing, generating low-ploidy cells that ultimately reduced the time required for the complete segregation of engineered lines.

## Introduction

*Synechococcus elongatus* PCC 7942, hereafter *Synechococcus* 7942, previously called *Anacystis nidulans* (Grigorieva and Shestakov, 1979), is a model freshwater cyanobacterium. It is a photoautotrophic, unicellular cyanobacterium with a genome of approximately 2.7 Mb and two endogenous plasmids, pANL and pANS (Van der Plas et al., 1992; Holtman et al., 2005; Chen et al., 2008). Throughout years of laboratory domestication, *Synechococcus* 7942 appears to have lost behaviours that are important in a natural environment, such as biofilm formation and phototaxis (Yang et al., 2018). *Synechococcus* 7942 has been used as a model strain for the analysis of photosynthesis and circadian rhythms (Atsumi et al., 2009; Xu et al., 2013), and it is one of the most common strains that are engineered to produce valuable biochemicals (Rubin et al., 2015; Ei and Ct, 2016; Kato et al., 2017). Fast growth rate, genetic tractability, and cost-effective cultivation are a few of the reasons to make this model strain attractive for genetic manipulation (Broddrick et al., 2016; Yin et al., 2019). Exogenous genes can be introduced into this strain *via* natural transformation. The transgenes can be delivered on independently replicating plasmids (Mermet-Bouvier et al., 1993) or integrated into the host genome *via* site-specific double homologous recombination (Liang et al., 2018). Although this mechanism is extensively used to create mutants, and *Synechococcus* 7942 possesses superior transformation properties compared to other model strains such as *Synechococcus* sp. PCC 6301 (Golden et al., 1989), the introduction of mutations across all genome copies is a time-consuming task because of the presence of multiple genome copies (polyploidy) in cyanobacteria.

The ploidy level of cyanobacteria is highly variable and influenced by the growth phase, growth rate, nutritional conditions, and stress (Zerulla et al., 2016; Ohbayashi et al., 2019; Pope et al., 2020; Riaz et al., 2021). Most cyanobacteria, including *Synechococcus* 7942, possess multiple genome copies irrespective of the growth rate or condition (Griese et al., 2011; Ohbayashi et al., 2019). Several studies have discussed the benefits of polyploidy in bacteria (Ludt and Soppa, 2019), but a major practical disadvantage of polyploidy is that it hinders the construction of mutants. There are many factors controlling the generation of fully segregated mutants, which include cyanobacterial strain background, lab conditions, and types of mutations (Lea-Smith et al., 2016). Compared to heterotrophic bacteria, the growth rate of cyanobacteria is slow (Burnap, 2015; Jaiswal et al., 2022), especially in solid medium (Hayashi et al., 2011; Gao et al., 2020), which further confers a challenge for cyanobacterial transformation. Previous studies have tried to determine the optimum conditions for the growth of multiple cyanobacteria on solid media. Agar substitutes, agar autoclaved separately from the salts in the medium, and the addition of thiosulfate have been used for multiple cyanobacteria (Thiel et al., 1989), reduced agarose concentration for axenic *Microcystis* strains (Shirai et al., 1989), and co-culturing of cyanobacteria with heterotrophs (Morris et al., 2011).

As genetically modified cyanobacteria could be used to produce low-cost bulk biochemicals (Andreas Angermayr et al., 2015) and high-value pharmaceuticals (Vijayakumar and Menakha, 2015), improvements in the previously existing gene transfer protocol could facilitate advances in cyanobacterial metabolic engineering. Current studies have tried to improve the natural transformation protocol by reducing the ploidy level (Pope et al., 2020) or increasing the antibiotic selection pressure (Nies et al., 2020). However, an improved segregation protocol for *Synechococcus* 7942 in this regard is not available. In this study, we have tested the feasibility of an improved segregation process for *Synechococcus* 7942. First, we selected several temperature and nutritional conditions and tested them using flow cytometry for the presence of miniploid cells, i.e. cells possessing the lowest possible number of genome copies. Low ploidy cells resulting from the high temperature and phosphate limitation were tested for their potential to produce fully segregated mutants in a shorter time. Solid medium culture conditions were also optimised using different concentrations of agar and the supplementation of antioxidants. In addition, the effect of antibiotic selection pressure on ploidy and segregation was also analysed with PCR. We identified that 0.35% agar concentration and 200 IU of catalase could improve the growth of *Synechococcus* 7942 on a solid growth medium, and overexpression of the catalase gene can help diluted cultures grow. Our findings suggest that higher temperatures, especially in a suspension culture and phosphate-depleted cells, produce better-segregated mutants in less time.

## Materials and methods

### Selected strains and culture conditions

The model cyanobacterium *Synechococcus elongatus* PCC 7942 (hereafter *Synechococcus* 7942) was purchased from the Pasteur Culture Collection of Cyanobacteria. The strain was routinely maintained in a conventional blue-green algae culture medium (BG-11) (Stanier et al., 1971) at 30°C. Preculture was grown in BG-11 until the early stationary phase. Constant agitation (100 r.p.m.) and constant illumination of 70 μmol m^–2^ s^–1^ were provided throughout the cultivation. Early stationary phase precultures were diluted to an optical density of OD_730_ of 0.05 for test conditions, e.g., increasing temperatures (22°C, 30°C, and 38°C), different concentrations of phosphates (0.92 mM and 0.023 mM), the addition of 100 mM bicarbonate, and different concentrations of phosphate with carbonates. Cells were collected from the mid to late log phase. Details are provided in Table 1. A solid BG-11 growth medium was prepared using agar powder (Solarbio, Beijing, China). 2X BG-11 and 2X agar suspension were autoclaved separately and mixed together after reaching ∼60°C to make a 1 × working medium. Catalase (Merck, Kenilworth, NJ, United States) and pyruvate (Solarbio, Beijing, China) were prepared in phosphate-buffered saline (PBS) or distilled water, respectively, and the filter was sterilised before addition to the medium.

**TABLE 1:** Selected cultivation temperatures and varying concentrations of carbonates and phosphates were used for the analysis.

Test condition	K_2_HPO_4_ (mM)	NaHCO_3_ (mM)	Temperature (°C)
Conventional BG-11	0.230	0	22, 30, 38
High carbonate (CC)	0.230	100	30
High phosphate (HP)	0.920	0	30
High phosphate and carbonate (HPC)	0.920	100	30
Low phosphate (LP)	0.023	0	30
Low phosphate with carbonates (LPC)	0.023	100	30

### Generation of *Synechococcus* 7942 transgenic strain overexpressing KatG gene and ΔglgA mutant

The self-replicative plasmid pLJD40 was constructed based on pLJD31, originating from the backbone of pAM5057 (Ma et al., 2014) and modified with the exchange of the antibiotic resistance gene to kanamycin. Plasmid pAM5057 was a gift from Susan Golden (Addgene plasmid # 120085^1^; RRID:Addgene_120085). The plasmid was constructed from three fragments using the pAM5057 backbone, with the kanamycin resistance gene amplified from pLJD31 and PsbA promoter and the 7942 catalase gene amplified from the genomic DNA of the 7942 strain. The plasmid was assembled using the Vazyme ClonExpress II one-step cloning kit (Vazyme, Nanjing, China). The corresponding oligonucleotides used for the construction of this plasmid are summarised in Table 2. The constructed plasmid was transformed into *E. coli* DH5α and reconfirmed by colony PCR and sequencing with MDLJCSTR495 and MDLJCSTR496 primer pairs. glgA- was a gift from Erin O’Shea (Addgene plasmid # 101841^2^; RRID:Addgene_101841) (Puszynska and O’Shea, 2017). For its transformation into *Synechococcus* 7942, we used the natural transformation protocol as described below.

**TABLE 2:** Oligonucleotides used in the study.

Name	Sequence 5′ to 3′	Description
MDLJCSTR159	ATGAGCCATATTCAACGGGAAAC	ΔGlgA diagnostics
MDLJCSTR160	TTAGAAAAACTCATCGAGCATCAAA	
MDLJCSTR161	ATGAGCCATATTCAACGGGAAAC	Amplification of Kan*^R^*
MDLJCSTR162	TTAGAAAAACTCATCGAGCATCAAA	
MDLJCSTR493	ATCGCTTTGATCTGGACTAAGCAGGAGAAGGCCATCCTGACGGAT	Amplification of plasmid backbone from pLJD31
MDLJCSTR494	AATCCAGCTGAGCGTAGGAAATCCCTGGGTTATTGGCCGA	
MDLJCSTR495	CCTACGCTCAGCTGGATTTAGCGTC	Amplification of PsbAII promoter from 7942 gDNA
MDLJCSTR496	TCCTTCAGTACTAGTACTAAAAACTCTTGCTTTTTAGG	
MDLJCSTR497	AGTACTAGTACTGAAGGAGGATATCCATATGACAGCAACTCAGGGTAAAT	Amplification of 7942 catalase gene from 7942 gDNA
MDLJCSTR498	TTAGTCCAGATCAAAGCGATCGGCA	

### Estimation of genome copy number

The distribution of genome copy numbers in populations of *Synechococcus* 7942 was determined using flow cytometry as described previously (Pope et al., 2020; Riaz et al., 2021). Briefly, around 5 × 10^7^ cells were collected from each test condition during the mid to late growth phase (OD_730_: 0.4–0.5) by centrifugation and washed two times with PBS. Cells were fixed with 70% ethanol and stored in the dark at −20°C for up to 3 weeks. Ethanol-fixed cells were washed two times with PBS and incubated with RNase A (1 μg/ml) at 37°C for 1 h. Cells were stained with propidium iodide (Sangon Biotech, Shanghai, China) following the manufacturer’s instructions. The 96-well flat-bottom plates were loaded with 200 μl of stained cell samples. In each case, 50,000 propidium iodide stained cells were analysed using an Attune NxT Flow Cytometer. Analysis of the data was performed on FlowJo v.10. To analyse the ploidy level by spectrofluorometer, the DNA amount was quantified using the Quant-IT dsDNA HS Assay kit (Invitrogen, Carlsbad, CA, United States) as described before (Riaz et al., 2021). Briefly, 5 × 10^7^ cells were collected by centrifugation and resuspended in distilled water to obtain a final OD_730_ of 0.2. An automated cell counter (IC1000; Countstar, Shanghai, China) was used to count the cells in suspension. Cells were disrupted using 0.75 g of zirconium beads in a bead beater (Shanghai Jingxin Technology, Shanghai, China) at 70 Hz for 2 min. The efficiency of cell breakage was verified by microscopy. The resulting suspension after cell lysis was centrifuged, and the supernatant was used for spectrofluorometric quantification (RF-5301PC; Shimadzu, China) at an excitation/emission of ∼502/523 nm using a Quant-IT dsDNA HS assay kit (Invitrogen, Carlsbad, CA, United States). A volume of 5 μl of assay reagent was mixed with 995 μl of assay buffer, and 2–20 μl of the sample was mixed with 200 μl of the prepared buffer. To plot a standard curve between the DNA concentration and the absorbance, 2 μg–100 μg of Quant-iT dsDNA HS standard were used. Genome copies were calculated using a standard curve plotted between known DNA amounts and fluorescence. Three biological replicates from each test condition were analysed.

### Natural transformation

For natural transformation, pure culture was grown at 30°C and 38°C in conventional BG-11 or low phosphate (LP) BG-11 (containing 0.023 mM K_2_HPO_4_) at 30°C. Cells were collected from mid to late log phase cultures (OD_730_ < 0.5). Enough culture was centrifuged to obtain an OD_730_ equivalent to 10^9^ cells. Collected cells were pelleted and resuspended in 100 μl of conventional BG-11 (BG-11 without phosphate for LP culture) and mixed with 500 ng of Δ*glgA* gene deletion construct (Addgene, plasmid#101841). Culture tubes were incubated at 30°C or 38°C in the dark for 12 h (mixing every 2 h). After 12 h, nearly 10–20 ml of fresh medium (without phosphate BG-11 in LP culture) was added without antibiotic and re-incubated for another 12–24 h. The culture was centrifuged and spread on the 10 μg/ml kanamycin (Kan) BG-11 plates (LP plates for LP culture). In liquid BG-11, 10 μg/ml Kan was added to grow suspension culture, and a 2-fold dilution in the same medium was prepared; 0.35% agar was used to prepare all solid growth media. After 8 days, cells were re-cultured onto 20 μg/ml Kan BG-11 plates.

### Chemical transformation of *Synechococcus* 7942

A pure culture of *Synechococcus* 7942 was grown in conventional BG-11 at 38°C. Cells were collected from mid-log phase cultures (OD_730_ < 0.5). Enough culture was centrifuged to obtain an OD_730_ equivalent to 10^9^ cells. Collected cells were pelleted, placed on ice, washed with ice-cold 0.05M CaCl_2_, and incubated for 30 min on ice. Pelleted cells were incubated with 500 ng of plasmid and 100 μl of 0.05M CaCl_2_ on ice. After 30 min, cells were immediately put into a water bath (42°C) for 2 min for heat shock and were put back into ice for recovery. Nearly 10–20 ml of fresh BG-11 medium was added without antibiotic and re-incubated for 12–24 h either at 30°C or 38°C (depending on the type transformation protocol). The culture was then centrifuged and spread on the 10 μg/ml Kan BG-11 plates.

### Genotyping

Individual colonies were mixed in distilled water, heated at 95°C for 3 min, centrifuged, and the supernatant was used for colony PCR. Transformation of pAM5057 plasmid was confirmed by amplification of the full kanamycin resistance gene using primer pair MDLJCSTR161 and MDLJCSTR162. Genome integration of Δ*glgA* gene deletion constructs and segregation of mutation was confirmed using primer pair MDLJCSTR159 and MDLJCSTR160.

### Validation of *Synechococcus* 7942 transgenic strain overexpressing KatG gene by catalase test and ΔglgA mutant using high performance liquid chromatography

A fresh culture of *Synechococcus* 7942 was mixed with 3% hydrogen peroxide in a tube or glass slide, and rapid bubble formation was observed (Taylor and Achanzar, 1972).

The glycogen content in WT and Δ*glgA* mutants was determined as described previously (Ernst et al., 1984; Porcellinis et al., 2017). Briefly, reference glycogen was prepared in 50 mM sodium acetate buffer and diluted to 10, 20, 40, 60, and 80 μg/ml in the same buffer. An amount of 15 mg of the dry weight of *Synechococcus* 7942 (mid-log phase) was mixed with 400 μl of 50 mM sodium acetate buffer. A volume of 400 μl of reference and sample were mixed with 200 μl of 0.333 mg/ml amyloglucosidase prepared in 50 mM sodium acetate buffer and 200 μl of 2 U/mL α-amylase prepared in 50 mM sodium acetate buffer. Reference and samples were heated at 60°C for 2 h and subjected to high performance liquid chromatography (HPLC) (Column: Agilent Hi-plex H). Oven temperature 60°C, RID detector, 0.0005M H_2_SO_4_ as flow phase, and 0.6 ml/min flow speed were used. Glycogen contents (as glucose) were calculated from the calibration curve (μg/ml).

## Results

### Effects of agar concentration, plating method, and additions of reactive oxygen species scavengers on cyanobacterial growth on solid media

**FIGURE 1:**
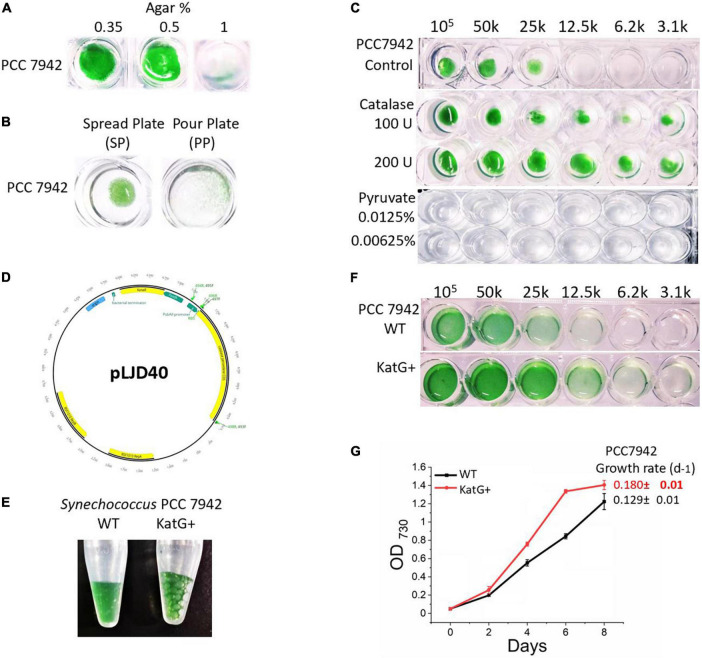
Cultivation optimisation of *Synechococcus* 7942. **(A)** Growth of *Synechococcus* 7942 on different agar concentrations. The culture was grown for 10 days at 38°C. **(B)** Comparison of spread plate (SP) and pour plate (PP) plating technique on *Synechococcus* 7942; 0.35% agar containing BG-11 was used in both plating techniques. **(C)** Effect of antioxidant (external catalase) on the diluted inoculum of *Synechococcus* 7942; 0.35% agar containing BG-11 was used as the control, while 100 IU or 200 IU was added to 0.35% agar containing BG-11 for test conditions. **(D)** Plasmid map of a self-replicative vector pLJD40. KatG genes are shown in green, and Kan resistance genes are shown in yellow. **(E)** A catalase test result of WT and KatG^+^ strains of *Synechococcus* 7942. **(F)** Effect of catalase on the diluted inoculum of WT and KatG^+^ strains of *Synechococcus* 7942. **(G)** Growth curve and growth rate per day of WT and KatG^+^ strains of *Synechococcus* 7942.

Cyanobacterial cultivation and the generation of mutants are challenging due to their slower growth rate (Hayashi et al., 2011; Burnap, 2015; Gao et al., 2020). A very concentrated inoculum is required to grow cyanobacteria on a solid medium. Generally, 1–1.5% agar is used as a solidifying agent (Allen, 1968). We cultivated nearly 10^6^ *Synechococcus* 7942 cells onto BG-11 containing different concentrations of agar (0.35%, 0.5%, and 1%). In addition, two different plating techniques, the surface plate method (SP) and the pour plate method (PP), were tested using 0.35% of agar. Plates were incubated for 10 days at 38°C. Results indicate that with the increasing concentration of agar, a decrease in growth occurs (Figure 1A). Cultivation on 0.35% and 0.5% of agar containing BG-11 medium showed significantly improved growth compared to 1% agar concentration. We conclude that 0.35% agar surface is hard enough to carefully perform streaking or spreading of bacteria, and this concentration promotes the growth compared to higher agar concentrations. The SP culturing method for *Synechococcus* 7942 was found superior to PP, and PP cultures produced pinpoint colonies, considering that it is hard to take out small merged colonies from the agar (Figure 1B).

Reactive oxygen species (ROS) are generated when autoclaving phosphates with agar (Tanaka et al., 2014). Catalases (Nantapong et al., 2019) and pyruvate (Kładna et al., 2015) are sometimes used in culture media as reactive oxygen species (ROS) scavengers. Catalase (100 IU and 200 IU) and pyruvate (5 mM and 1 mM) were added to BG-11 0.35% agar media to test their effect on cell growth. The addition of 100 IU and 200 IU of catalase promoted the growth of *Synechococcus* 7942 in diluted inoculum on a solid growth medium compared to control, as shown in Figure 1C. As an alternative ROS scavenger, 10 mM pyruvate was used (Gyulkhandanyan et al., 2003). However, even the low concentrations (1 mM) completely inhibited the growth of *Synechococcus* 7942; hence, it was not used for subsequent experiments. It is concluded that the reduction in agar concentration positively affects the growth of diluted inoculum, and the addition of catalase can improve it further.

### Overexpression of the catalase gene enhances cyanobacterial growth

Cyanobacteria grow better in liquid BG-11 compared to the solid medium. However, their overall growth rate is lower compared to heterotrophs. As *Synechococcus* 7942 possess a single gene for catalase-peroxidase, and the addition of external catalase improved the growth (Figure 1), it was speculated that ROS could be one of the reasons for their slower growth in solid medium (Gao et al., 2020). Hence, in an attempt to increase the growth rate, a catalase-peroxidase (katG) gene was amplified, cloned, and expressed (Figure 1D). Verification of transformation was done by PCR amplification of the whole kanamycin gene, while the functionality of catalase gene expression was verified by the addition of hydrogen peroxide (H_2_O_2_) onto the fresh culture of wild type (WT) and transgenic strain overexpressing the katG gene (KatG^+^) strain. Rapid bubble formation was observed in positive clones compared to the WT (Figure 1E). When WT and KatG^+^ strain was grown with decreasing concentration of inoculum, a more diluted culture of transgenic strain was able to grow compared to WT, in addition, significantly enhanced growth rate per day (0.129 ± 0.01 to 0.180 ± 0.01) (*t*-test: *P* < 0.05), implying that catalase overexpression can increase growth by reducing oxidative stress (Figures 1F,G).

### An increase in temperature and decrease in phosphate produces most miniploid cells

**FIGURE 2:**
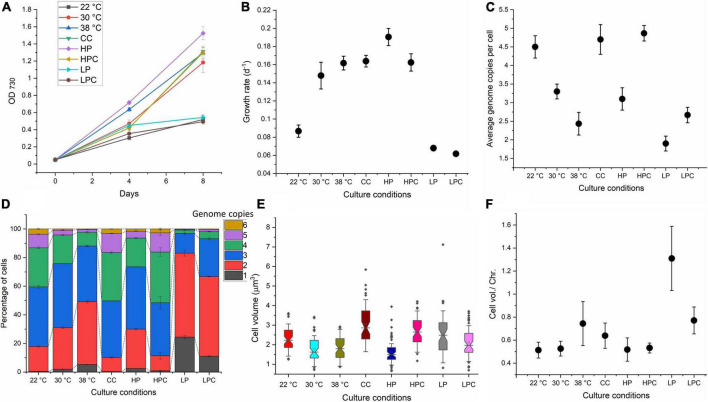
Ploidy estimation of *Synechococcus* 7942 under different temperature and nutritional conditions. **(A)** Growth curve of *Synechococcus* 7942 at different tested conditions. Samples for the analysis were taken from all conditions on day 4 (OD_730_: 0.4–0.5), which was the mid or late log phase. **(B)** The specific growth rate of *Synechococcus* 7942 at different tested conditions. **(C)** The average genome copies per cell under all tested conditions. **(D)** Distribution of genome copies among the populations of *Synechococcus* 7942 under different tested conditions. **(E)** Cell volume (μm^3^) under all tested conditions was determined using ImageJ. **(F)** Cell volume per chromosome (μm^3^) was determined by dividing average cell volume with the ploidy level under test conditions. High carbonates culture (CC); high phosphate culture (HP); high phosphate and high carbonate (HPC); low phosphate (LP), and low phosphate and high carbonate (LPC).

Cyanobacteria are polyploid, and the introduction of mutations into all genome copies remains a time-consuming task. Several test conditions were selected in this study to identify the culture condition that could produce the maximum proportion of miniploid or diploid cells (Table 1). Three different culture temperatures, namely, 22°C, 30°C, and 38°C, were tested. A previous study suggested that an increase or decrease in temperature results in ploidy fluctuation in *S. elongatus* (Ohbayashi et al., 2019). In accordance with this study, an increased temperature resulted in an increased growth rate (Figures 2A,B) and a reduction in ploidy level, such as 4.5 ± 0.3 average genome copies at 22°C, 3.3 ± 0.2 at 30°C, and 2.4 ± 0.3 at 38°C were recorded (Figure 2C). Flow cytometry also revealed more miniploid (∼5%) and diploid cells (∼44%) at 38°C compared to 30°C and 22°C (Figure 2D). Cell volume and cell volume per chromosome were reduced at lower temperatures (Figures 2E,F), implying that some temperature-associated factors regulate ploidy in *Synechococcus* 7942. These results suggest that increasing the temperature may lower the culture growth time and generate a larger proportion of miniploid cells that could facilitate the generation of segregated mutants. Hence, we have used log-phase cultures at 38°C to check their potential for rapid segregation.

We have further examined the effects of different nutrients on the growth rate, ploidy level, and cell volume at constant temperature (30°C). In conventional BG-11, 100 mM sodium bicarbonate (NaHCO_3_) was added as a source of CO_2_ (termed CC) as previous studies have reported the increase in growth of cyanobacteria with NaHCO_3_ supplementation (Sarma and Garg, 1985). Genome copy number largely depends on the availability of phosphate. Varying phosphate concentrations (K_2_HPO_4_), 4 times higher amount of K_2_HPO_4_ (HP) and 10 times lower amount of K_2_HPO_4_ (LP), were tested to observe the effect of abundance or limitation of phosphates. Moreover, 100 mM NaHCO_3_ was supplemented with HP and LP cultures to produce HPC and LPC cultures. The results are summarised in Figure 2. The availability of carbonates increased the growth rate, ploidy level, and cell volume. In contrast to the elevated temperature, a higher growth rate in CC was accompanied by a higher ploidy level, while the highest growth rate was observed in HP cultures yet maintained a similar ploidy (3.1 ± 0.3) to the control (30°C). The ploidy level as well as the growth rate of LP cultures were reduced (1.9 ± 0.2) and produced the highest proportion of miniploid and diploid cells. Interestingly, the highest cell volume per chromosome was observed in LP cultures. These results suggest that phosphate-depleted cells possess the least number of genome copies, and thus, were selected for transformation.

### Suspension culture at elevated temperature and phosphate depleted cells provide rapid mutation segregation

**FIGURE 3:**
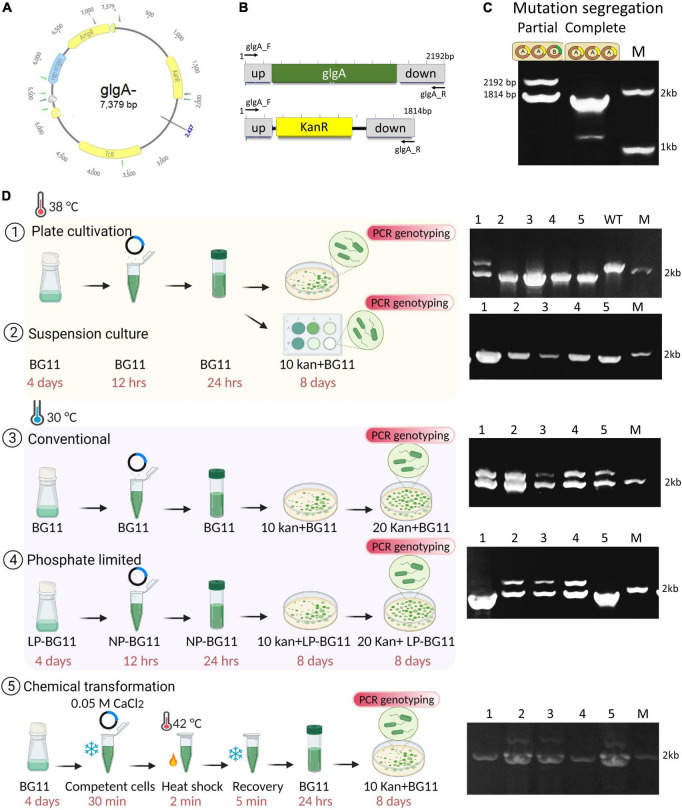
Transformation of *Synechococcus* 7942 with Δ*glgA* gene deletion plasmid. **(A)** Map of the Δ*glgA* gene deletion construct obtained from Addgene (plasmid#101841). **(B)** The integration site and primer binding sites are coloured grey, the *glgA* gene is shown in green, and the kanamycin (Kan) resistance gene is shown in yellow. **(C)** Agarose gel for the PCR with the primer pair (glgA_F (MDLJCSTR159) and glgA_R (MDLJCSTR160) shows complete and partial transgene segregation. **(D)** Schematics of transformation protocol and validation of mutants by PCR genotyping. Cultures of *Synechococcus* 7942 used for transformation were taken from three conditions; 38°C, 30°C, and phosphate-limited BG-11. Suspension culture and traditional plating methods of transformation were tested. Validation of segregated mutants was performed using the primer pair glgA_F and glgA_R. High temperature produced most of the fully segregated mutants. No fully segregated mutants were obtained at 30°C. Phosphate-depleted cultures produce nearly 50% of segregated mutants. Chemical transformation of *Synechococcus* 7942 was successfully performed using 0.05 M CaCl_2_. Created with BioRender.com.

Ploidy estimation findings suggest that a reduction in ploidy occurs when the temperature is increased, or the amount of phosphate is decreased 10-fold. We reasoned that this could reduce the time to get fully segregated mutants. Using Δ*glgA* deletion plasmid, the natural transformation was performed, and positive clones were validated by amplifying the upstream and downstream regions of the *glgA* gene (Figures 3A–C). The Δ*glgA* mutants were also validated by the detection of glycogen. An amount of 15 mg of WT dry biomass showed a glycogen level of 196 ± 26 μg/15 mg of dry biomass, while no glycogen was detected in fully segregated mutants.

We have selected three different culture conditions (30°C, 38°C, and LP culture) and two different transformation protocols. One utilises the suspension culture, and the other is the traditional plating method. The results are summarised in Figure 3D. An increase in temperature produced more mono and diploid cells. After the first culturing, segregation of mutations was complete (nearly 80% of clones) using the traditional plating method compared to the culture at 30°C where segregation was incomplete even after two subculturings (Figure 3D). To further confirm the effect of temperature on mutation segregation and test the suspension culture method (instead of the conventional plating method), the whole mixture of plasmid and cells was added to 10 μg/ml of kanamycin-containing liquid BG-11 medium. PCR genotyping was performed using 1 ml culture from each dilution. Remarkably, results showed that all of the transformed cells were fully segregated, implying that the suspension culture method could be preferred to get the maximum number of cells possessing fully segregated mutations (Figure 3D).

Low phosphate culture produced ∼20% of miniploid cells and nearly 60% of diploid cells. Although the reduction in phosphate slows down the overall growth rate, the initial growth rate until the cells reach the OD_730_ of 0.4 is comparable to its respective control (30°C). We have used OD_730_ around 0.4 for transformation, which is considered a log phase. After this OD, growth becomes slow, presumably due to phosphate starvation, and cells completely stop growing after reaching an OD_730_ of 0.6–0.7. As expected, nearly 50% of clones were fully segregated. If a high cultivation temperature is not desired, phosphate-depleted cells are a suitable method to accelerate the obtention of fully segregated clones.

### Chemical transformation of *Synechococcus* 7942

In addition to natural transformation, *Synechococcus* 7942 was tested for its potential for chemical transformation, as chemically competent cells can be stored for a longer time compared to natural transformation, which requires freshly grown log-phase cultures (Figure 3D). Different salts, e.g., CaCl_2_, MgCl_2_, RbCl_2_, and lithium acetate, were tested, and 0.05 M CaCl_2_ was found to be the most effective. The number of colonies obtained after 0.05 M CaCl_2_ (134 ± 38) and 0.05 M MgCl_2_ (8 ± 2) transformations was significantly lower than that of the natural transformation (27 × 10^2^). The remaining two conditions did not result in successful transformation. Despite this shortcoming, a sufficient number of clones possessing complete segregation was obtained in calcium chloride treatment (Figure 3D).

### Ploidy level, antibiotic selection pressure, and mutation segregation

**FIGURE 4:**
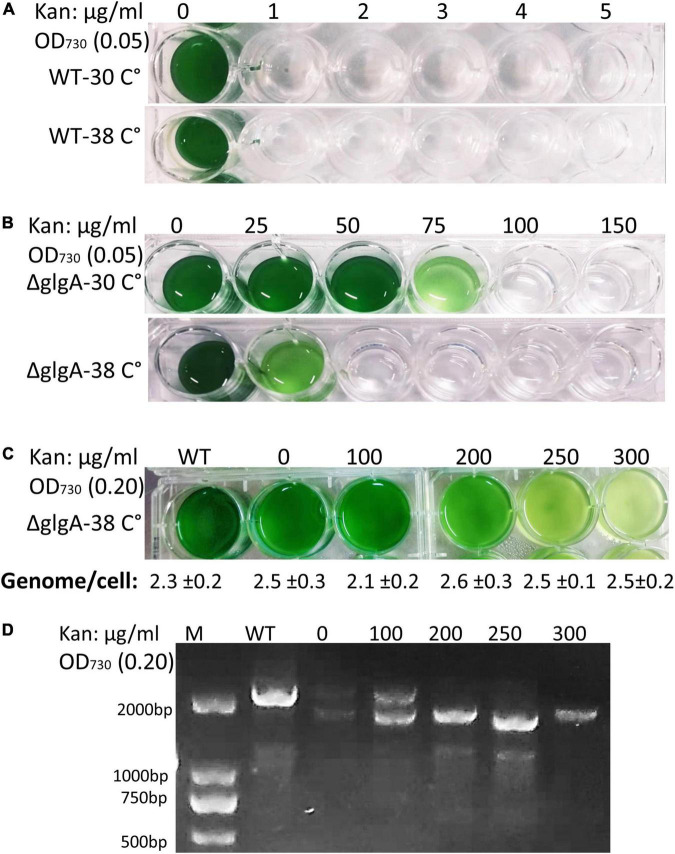
Effect of different concentrations of kanamycin on the growth, genome copies, and mutation segregation in *Synechococcus* 7942. **(A)** Wild-type (WT) cells were unable to grow even at 1 μg/ml of kan concentration at any tested temperature (30°C or 38°C). Initial OD_730_: 0.05. **(B)** Transformed cells (Δ*glgA*) were able to grow at a higher concentration of Kan (75 μg/ml) at 30°C, while the same number of cells was unable to tolerate Kan concentration higher than 25 μg/ml at 38°C. Initial OD_730_: 0.05. **(C)** Transformed cells (Δ*glgA*) grew at a higher concentration of Kan when a higher initial inoculum (OD_730_: 0.20) was used compared to initial OD_730_: 0.05. No significant effect of antibiotic concentration on genome copy number was observed. **(D)** A higher concentration of Kan (above 100 μm/ml) promotes the selection of homozygous transformants.

Antibiotic concentration in the medium affects the selection of homozygous transformants, allowing better-segregated mutants to survive at higher antibiotic concentrations. Previous studies have shown that transformants are capable of surviving at a very high concentration of Kan (8.3 mg/ml) (Nies et al., 2020). Our results have shown that WT cells were unable to survive 1 μg/ml at either 30°C or 38°C (Figure 4A). When a fully segregated colony (initial OD_730_: 0.05) of the Δ*glgA Synechococcus* 7942 was cultured in an increasing concentration of kanamycin, it was not able to survive at 50 μg/ml at 38°C while it could survive the Kan concentration of 75 μg/ml at 30°C (Figure 4B). As the ploidy level was higher at 30°C than at 38°C, it contributed to higher antibiotic resistance, presumably due to the higher copy number of the selection marker. Genome copies were determined by growing transformed cells of *Synechococcus* 7942 at different antibiotic concentrations. No effect of the antibiotic on genome copies was observed at a similar temperature (Figure 4C). However, higher antibiotic concentrations affect the selection of homozygous transformants, and fully segregated clones were obtained at very high concentrations of Kan (Figure 4D).

## Discussion

Agar-based growth medium has been used for bacterial culturing, isolation, and purification for over 120 years. However, there has always been an inconsistency between actual and cultivable cell counts on plates. This phenomenon is often referred to as “a great plate count anomaly”, which is still unsolved (Tanaka et al., 2014). Previous study has also suggested the inhibitory effects of agar on the growth of cyanobacteria (Allen and Gorham, 1981). Effective generation of mutants in cyanobacteria becomes more challenging due to inadequate growth of cyanobacteria on agar medium compared to heterotrophic bacteria (Gao et al., 2020). We found that 0.35% of agar concentration encourages the growth of cyanobacteria and submerged agar colonies were pinpointed and growth was suppressed. Previous study on a thermophilic cyanobacterium showed that *Thermosynechococcus elongatus BP-1* produces small colonies if cells are merged inside agar, while on the surface of the agar, colonies were large (Iwai et al., 2004). In addition, the number of clones was also increased in merged cultures of *Thermosynechococcus elongatus BP-1.* One possible explanation for getting more colonies (in submerged cultures) in previous studies may be large because of the concentration of agar, not the plating technique. For surface culture, an increased agar concentration was used (1%), and for submerged cultures, cells were embedded in a thin layer of 0.35% agar. It was speculated that embedded cultures had reduced colony size because of the increased resistance of the motile cells to make spreading colonies; however, *Synechococcus* 7942 is non-motile and still produces pinpoint colonies in pour plate cultures. The higher diffusion rate in less concentrated agar medium is supposed to affect pH, nutrient supply, and accumulation of waste and could be a controlling growth factor in semisolid culture medium.

Accumulation of ROS in the culture medium suppresses the growth of microbes, which may prevent the growth of transformants that are already facing antibiotic selection pressure. Many cyanobacterial genomes lack catalase genes, e.g., *Prochlorococcus* (Morris et al., 2011) and *Thermosynechococcus* E542 (Liang et al., 2019). *Synechococcus* 7942 possesses a single gene for catalase-peroxidase, while other bacteria contain multiple catalase isoenzymes (Barrière et al., 2002) that give rapid response upon the addition of hydrogen peroxide. Catalase overexpression in *Synechococcus* 7942 played a significant role in promoting growth by overcoming ROS stress in this study. Hence, overexpressing catalase genes could eliminate the cost required for the addition of external catalase.

Ploidy level varies in cyanobacteria, and multiple factors influence ploidy level in cyanobacteria. Irrespective of the condition, cyanobacteria remain polyploid, which is a major obstacle to segregating mutations across all genome copies. Miniploid and diploid cells and cell volume per chromosome were reduced at lower temperatures (Figure 2), implying that ploidy in *Synechococcus* 7942 is regulated by some temperature-associated factors. Growth rate and ploidy level at constant temperature are not correlated in *Synechococcus* 7942 and are regulated by nutrient availability. CC culture possessed more genome copies and growth rate, while HP culture showed an increased growth rate yet maintained a similar ploidy level. This conclusion is also supported by the growth rate and ploidy level of LP cultures where the growth rate was low, and phosphate limitation reduced the ploidy to 1.9 ± 0.2 and produced the highest proportion of miniploid and diploid cells. *Synechococcus* 7942 seems to use DNA as a phosphate source (Zerulla et al., 2014) because phosphate limitation drastically reduces the ploidy level. Interestingly, the highest cell volume per chromosome was observed in LP cultures. Although genome copies and cell volume are positively correlated, the lowest ploidy cells bear large cell volumes, implying that phosphate deficiency reduces cell division but not cell growth (Li et al., 2016). This could also be an adaptive strategy for the cell to store the materials to resume cell division upon resupply.

Using miniploid cells (elevated temperature and LP cultures), we have developed a rapid protocol for natural transformation. At elevated temperatures, the same cells showed different potential for mutation segregation; for example, the efficiency of suspension culture to produce fully segregated clones was nearly 100% compared to the traditional plating method. We speculate that on a solid medium, bacterial cells experience a localised effect of the antibiotic; hence the selection pressure may be reduced; and in a liquid medium, this selection pressure is increased. Another possible explanation is that in the liquid medium, those cells have the most antibiotic selection pressure alongside reduced unknown agar inhibitory factors. The limitation of this method is that to get an individual clone, the liquid culture needs to be restreaked. Consequently, suspension culture is the rapid method that produces the most segregated mutants compared to other tested conditions.

## Conclusion

In this study, we have revisited traditional approaches to the transformation of the model cyanobacterium *Synechococcus elongatus* PCC 7942 and improved them through the combination of ploidy control, lowering the concentration of the solidifying agent, and application of reactive oxygen scavengers. The three-fold reduction of solidifying agent concentration, mitigation of reactive oxygen species with exogenous and endogenous catalases, and reducing the cellular ploidy to an average of 2–3 per cell all had a substantial and positive impact on the rapid generation and segregation of transformants. Furthermore, the elevation of the cultivation temperature to 38°C resulted in the rapid formation of miniploid cells for transformation. The proposed improved method of cyanobacteria transformation and rapid segregation can drastically accelerate the generation and full segregation of the desired transformants from a few weeks to several days.

## Data availability statement

The original contributions presented in this study are included in the article/supplementary material, further inquiries can be directed to the corresponding author.

## Author contributions

SR contributed to the conceptualisation, methodology, validation, formal analysis, investigation, data curation, writing – original draft and review and editing, visualisation, and supervision. YJ and MX contributed to the investigation and data curation. DY contributed to the investigation and resources. AK-S contributed to the resources, validation, formal analysis, writing – review and editing, visualisation, and funding acquisition. FR contributed to the methodology, validation, formal analysis, investigation, writing – review and editing, supervision, and project administration. MD contributed to the conceptualisation, methodology, resources, validation, formal analysis, investigation, data curation, writing – original draft and review and editing, visualisation, supervision, project administration, and funding acquisition. All authors contributed to the article and approved the submitted version.

## Conflict of interest

The authors declare that the research was conducted in the absence of any commercial or financial relationships that could be construed as a potential conflict of interest.

## Publisher’s note

All claims expressed in this article are solely those of the authors and do not necessarily represent those of their affiliated organizations, or those of the publisher, the editors and the reviewers. Any product that may be evaluated in this article, or claim that may be made by its manufacturer, is not guaranteed or endorsed by the publisher.

## Footnotes

1
http://n2t.net/addgene:120085

2
http://n2t.net/addgene:101841
